# Description of a new species of *Euderus* Haliday from the southeastern United States (Hymenoptera, Chalcidoidea, Eulophidae): the crypt-keeper wasp

**DOI:** 10.3897/zookeys.645.11117

**Published:** 2017-01-12

**Authors:** Scott P. Egan, Kelly L. Weinersmith, Sean Liu, Ryan D. Ridenbaugh, Y. Miles Zhang, Andrew A. Forbes

**Affiliations:** 1 Department of BioSciences, Rice University, Houston, Texas, 77005; 2 Department of Biology, University of Central Florida, Orlando, Florida, 32816; 3 Department of Biology, University of Iowa, Iowa City, Iowa, 52242

**Keywords:** Bassettia pallida, Chalcidoidea, Cynipidae, Euderus, Eulophidae, Quercus geminata, Quercus virginiana, new species

## Abstract

A new species of the genus *Euderus* Haliday, *Euderus set* **sp. n.**, is described and illustrated from the southeastern United States, where it parasitizes the crypt gall wasp, *Bassettia pallida* Ashmead, 1896, on live oaks in the genus Quercus (subsection Virentes). This is the 1^st^ species of the genus reported from the southeastern United States to parasitize cynipid gall wasps and the 3^rd^ species of the genus reported to attack cynipids in North America. Modified sections of the identification keys to subgenera and species of *Euderus* (Yoshimoto, 1971) are included to integrate the new species.

## Introduction

The genus *Euderus* Haliday, 1844 is a group of parasitic wasps in the family Eulophidae (Hymenoptera) with approximately 77 described species (Yoshimoto 1971, Noyes 2016). The genus has a cosmopolitan distribution, where it is found in North and South America, Europe, Asia, Australia, and many isolated island archipelagos, including the Hawaiian islands, Micronesia, the Canary Islands, and the Seychelles archipelago (Ahmad 1976, Askew et al. 2001, Báez and Askew 1999, Gates et al. 2002, Gibson et al. 2006, Goolsby et al. 2001, Gunasena and Harris 1998, Herting 1973, Thompson 1955, Yoshimoto 1971).

Yoshimoto (1971) produced the last revision of the genus *Euderus* for North America, north of Mexico. The revisions built off of Nearctic catalogues by Peck (1951, 1963) and Burks (1967), with new additions based on material deposited in the Canadian National Collection, material loaned from the Natural History Museum, London, Philadelphia Academy of Sciences, and the Museum of Comparative Zoology, Harvard University. Based on this revision, there are 22 species reported from North America north of Mexico, with 12 residing in the coastal southeastern U.S. (Florida, Alabama, Mississippi, Louisiana, and Texas; Yoshimoto 1971, Noyes 2016). According to Yoshimoto (1971), the biological records of *Euderus* from the U.S. indicate that most species are host specific on pupae of leaf tying, leaf mining, twig and fruit boring Lepidoptera (Tortricidae or Gelechiidae) and stem boring and other herbivorous Coleoptera (Buprestidae, Cerambycidae, and Curculionidae). Rarely, *Euderus* has been reported to attack gall-making Hymenoptera (Cynipidae) or exhibit hyperparasitism on other Hymenopteran parasitoids (Ichneumonidae). Detailed host records from field observation across the genus are summarized in Table 1 of Yoshimoto (1971).

Here, we report the first species of this genus from the southeastern United States to attack cynipid gall wasps, where it is associated with the crypt gall wasp *Bassettia pallida* Ashmead, 1896 (Hymenoptera; Cynipidae) on live oaks (Quercus; subsection Virentes), including *Quercus virginiana* and *Quercus geminata*. We modify the key published by Yoshimoto (1971) to include the new species and add a correction to the key to subgenera.

## Materials and methods

### Field collections and lab husbandry

For the type locality, branches of the sand live oak, *Quercus geminata*, infested with the asexual generation of the crypt gall wasp, *Bassettia pallida*, were collected July 15, 2014, August 1, 2015, and October 1, 2015, in Inlet Beach, Florida (Lat/Long: 30.273663, -86.001911). Additional populations were collected across the U.S. Gulf coast on *Quercus geminata* and *Quercus virginiana* in 2014, 2015, and 2016 (see Table 1). Branches were placed in clear plastic cups, covered with a coffee filter and rubber band, and maintained outside in natural temperature and humidity conditions in a constantly shaded walkway at Rice University in Houston, Texas (Lat/Long: 29.717030, -95.401279). Emergence of all individuals was monitored regularly for a year or dissected out of *Bassettia pallida* crypt galls in the lab. All individuals were preserved in 96% EtOH and frozen in a -80°C ultrafreezer.

**Table 1.** Confirmed localities for *Euderus set* associated with *Bassettia pallida* galls on live oaks. (LA = lab emergence from a *Bassettia pallida* gall; D = found during dissection of *Bassettia pallida* gall).

Location	Lat/Long	Host plant	Collection method	N
Inlet Beach, FL	30.273663, -86.001911	*Quercus geminata*	LA, D	158
Lake Lizzie, FL	28.227718, -81.179641	*Quercus geminata*	D	12
Ochlocknee Bay, FL	29.922913, -84.411060	*Quercus geminata*	D	7
Jekyll Island, GA	31.073975, -81.424541	*Quercus virginiana*	LA	1
Gautier, MS	30.382323, -88.611080	*Quercus virginiana*	D	3
Delcambre, LA	29.968115, -91.981863	*Quercus virginiana*	D	2
Morgan City, LA	29.693581, -91.159113	*Quercus virginiana*	D	1
Humble, TX	29.998392, -95.184455	*Quercus virginiana*	LA	19
Rice Univ., TX	29.716882, -95.401928	*Quercus virginiana*	LA, D	27

### Morphological descriptions and type material locations

Descriptions of the species have been made under a Leica M125 Stereoscope, with lighting achieved through a Leica LED5000 SLI - Spotlight illumination with 2 HiPower LEDs and a Leica TL5000 Transmitted Light Base with Rottermann Contrast TM, brightfield and two sided darkfield. For images, 75-150 stacked photographs were produced by a Canon 7D Mark II (Canon USA, Melville, NY), with a Mitutoyo M Plan Apo 10x objective mounted onto the Canon EF Telephoto 70 – 200mm zoom lens, which was mounted on a Stackshot Automated Focus Stacking Macro Rail (Cognysis Inc., Traverse City, MI). The Canon MT–24EX Macro Twin Lite Flash with custom made diffusers was used to minimize hot spots. Images were processed using Zerene Stacker (Zerene Systems LLC., Richland, VA) and plates were finished with Adobe Illustrator CC. Pictures of slide-mounted wings were taken using a Leica ICC50W camera.

Morphological nomenclature follows Gibson et al. (1997), Yoder et al. (2010), and Hymenoptera Anatomy Consortium (2016). The identification key is modified from the key to subgenera and species of the genus *Euderus* in Yoshimoto (1971).

Type material is deposited in the American Museum of Natural History (AMNH) (Curator: Dr. James Carpenter; Collection Assistant: Christine LeBeau). AMNH specimen identification codes: Holotype - AMNH_IZC 00238642; 8 paratypes - AMNH_IZC 00238643 – 00238650.

### Complementing morphological taxonomy with molecular barcodes

When samples were of sufficient quality for genetic work, we complemented morphological taxonomy with molecular barcodes (e.g., Smith et al. 2008, 2012, Forbes and Funk 2013, Forbes et al. 2016). Genomic DNA from two individuals from the Inlet Beach, FL population were extracted using DNeasy Blood and Tissue kits (Qiagen Inc., Valencia, CA). We used a pair of degenerate primers to amplify a segment of the mitochondrial cytochrome oxidase (mtCOI) gene using standard PCR protocols (Smith et al. 2008). Primers used were COI pF2: 5’ - ACC WGT AAT RAT AGG DGG DTT TGG DAA - 3’ and COI 2437d: 5’ - GCT ART CAT CTA AAW AYT TTA ATW CCW G - 3’, developed by Simon et al. (1994) and modified by Kaartinen et al. (2010). We treated amplified fragments with Exonuclease I (New England Biolabs, Ipswich, MA) and Shrimp Alkaline Phosphatase (Fermentas Life Sciences, Glen Burnie, MD) and sequenced in both forward and reverse directions on an ABI 3730 DNA analyzer using BigDye 3.1 sequencing chemistry (ThermoFisher Inc., Waltham, MA). We edited raw sequences and assembled forward and reverse reads using Geneious v.6.1.8 (Kearse et al. 2012). The final sequences were 703bp and 745bp in length, a function of amplification and sequencing success. We ran each sequence through the “identification request” module on the Barcode of Life Database (BOLD; Ratnasingham and Hebert 2007) to identify the highest percentage matches from previously identified taxa. All sequences were deposited in GenBank (accession numbers provided below).

## Results

Details on the on the type material and type localityare provided, with a thorough description with images, a differential diagnosis of the new species, and a modification to the identification key published by Yoshimoto (1971) that distinguishes this new species from closely related species. We also provide a correction to the key to subgenera published in Yoshimoto (1971). In addition, we provide a brief description of etymology and information on the distribution, natural history, biology, and results of the mtDNA DNA barcoding analysis. In Supplemental File 1, we provide further details of the morphological, geographic, and ecological distinguishing features of this new *Euderus* species, *Euderus set*, which differentiate it from (1) ecologically similar *Euderus* species attacking cynpid gall wasps, (2) geographically overlapping *Euderus* species in the coastal southeastern United States, (3) geographically proximate *Euderus* speices from the Caribbean, and (4) morphologically similar species within the *Euderus* genus. We also provide the DNA sequence data to complement the morphological taxonomy in Suppl. material 1.

### Taxonomy

#### Euderus

Taxon classificationAnimaliaHymenopteraEulophidae

Haliday, 1844

Euderus Haliday, 1844. Trans. Ent. Soc. Lond. 3: 298.

##### Type-species.

*Entedon amphis* Walker.

#### Euderus set

Taxon classificationAnimaliaHymenopteraEulophidae

Egan, Weinersmith, & Forbes sp. n.

http://zoobank.org/44A7E50B-5E80-4A60-9170-DD1B9A7221B7

**Figure 1.**
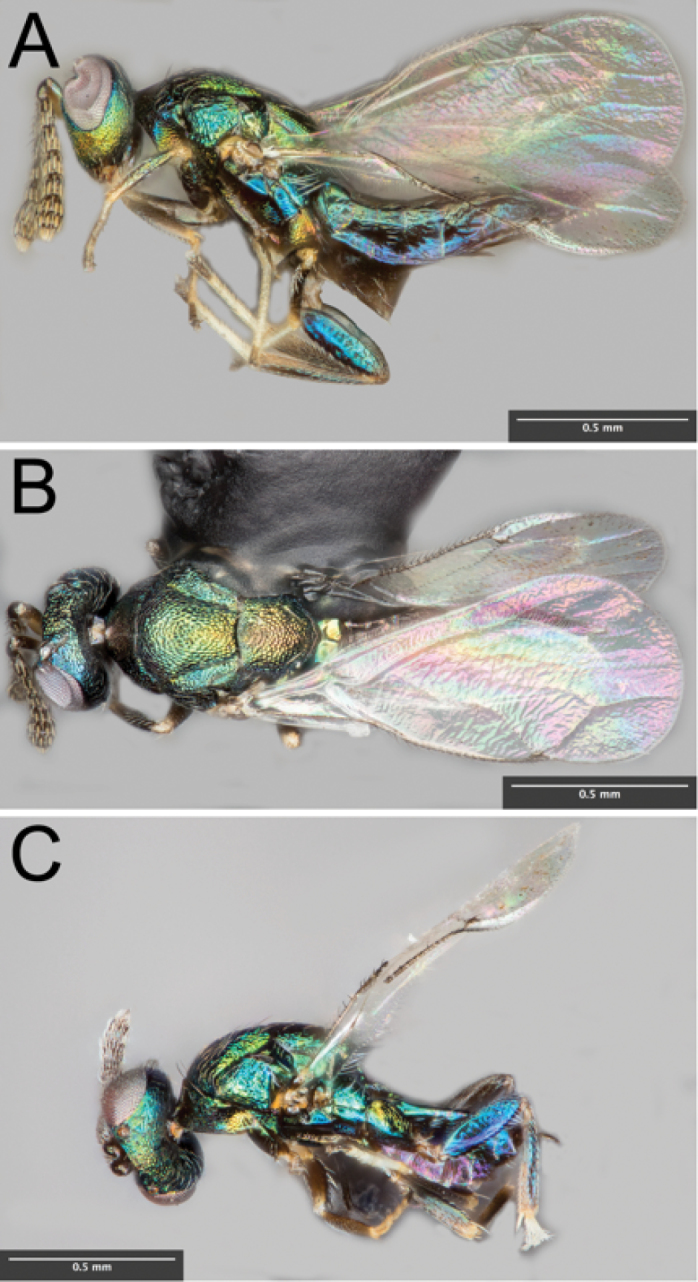
**A** Lateral habitus of female *Euderus set* **B** Dorsal habitus of female *Euderus set* **C** Lateral habitus of male *Euderus set*.

**Figure 2.**
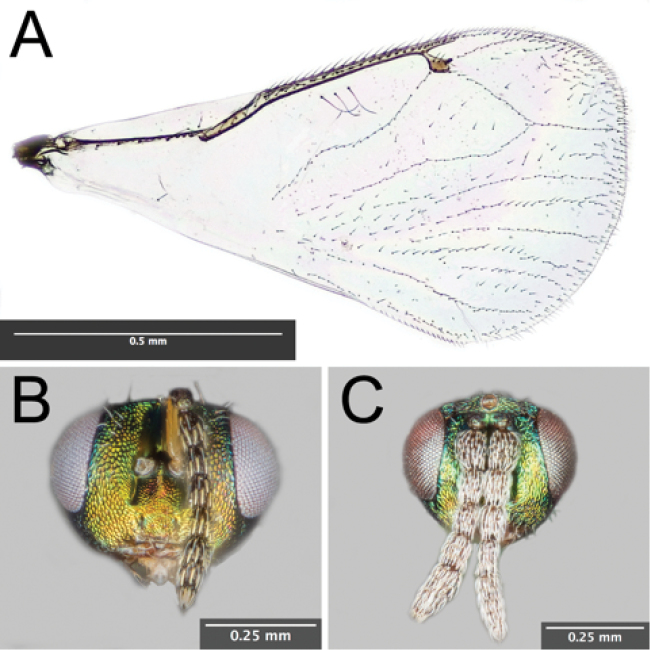
**A** Right forewing of male *Euderus set* **B** Anterior view of female *Euderus set* head **C** Anterior view of male *Euderus set* head.

Figs 1 , 2 (3 panels per figure)

##### Material examined.

Holotype, ♀, Inlet Beach, FL, collected July 15, 2015 by Scott P. Egan, AMNH ID#: AMNH_IZC 00238642

Allotype, same data as holotype

Paratypes, 2 ♂, same locality as holotype

Specimens from each collection locality (Table 1).

##### Description.

**FEMALE.** Length 1.6 – 2.3 mm. Holotype 2.3 mm

***Color*.** Head, scrobal depression, pedicel, flagellum, mesoscutum, scutellum, coxae and metasoma metallic, olive green to turquoise to iridescent blue (colors depends on lighting and age of specimen); antennal scape white to yellow; femora and tibiae concolorous with mesoscutum but color lightens apically; tarsi white, except terminal segment dark brown (Fig. 1A, B).

***Head*.** Head in fresh specimens as wide as mesosoma; in dorsal view 2.9 times as broad as long; eyes prominent and bare; vertex, frons, and clypeus reticulate; vertex and upper frons distributed with white bristles; scrobal depression extends from slightly below anterior ocellus to level of lower eye margin, smooth above torulus and striolate below; toruli located in lower third of scrobal depression; clypeus short, subquadrate, only slightly longer than wide; malus sulcus inconspicuous and 0.44 times eye length; mandibles with three teeth. Antennal scape 3.5 times as long as broad and 0.6 times eye length; Flagellum with nine segments, with anellus two-segmented, funicle four-segmented, and clava three-segmented. Funicular segments each with 2 rows of thick, mostly non-overlapping bristles (Fig. 2B). Relative length of scape, pedicel, anelli, funicle 1, 2, 3, and 4, and clava 1, 2, and 3 as 38, 12, 2, 24, 22, 22, 20, 16, 12, 8, respectively; two anelli with the same length but relative breadth of first anellus to second anellus as 6, 8.

***Mesosoma*.** Pronotum, mesoscutum and scutellum reticulate; pronotum short in dorsal view, with six brown bristles at margin with mesoscutum; mesoscutum sparsely setose and setae inconspicuous; scutellum with many short setae and two pairs of strong setae in posterior third. Mesoscutum 0.95 as long as broad, mid-lobe convex, notauli complete and deep; axillae slightly advanced, their anterior tip extending to the approximate midpoint of the mesoscutum. Scutellum moderately convex and length 0.80 times length of mesoscutum. Propodeum 0.24 times length of scutellum and with strong median carina (Fig. 1B); callus with 6–7 bristles (Fig. 2A).

***Wings*.** Forewing broad, extending past apex of gaster, marginal ciliae short; basal cell bare; submarginal vein with 6 dorsal bristles; postmarginal vein 1.3x length of stigmal vein; 3 admarginal hairs on left wing, four admarginal hairs on right wing; stigmal vein short and with large stigmus with 6 hairs on surface; 5 strongly-defined hair lines reaching dorsal and apical margin of forewing, with 3–4 additional less strongly defined hair lines that may or may not reach wing margin; 1 hair at median of radial cell (Fig. 2A). Hindwing 0.80 times length of forewing; hindwing moderately and evenly setose; hind marginal ciliae long.

***Metasoma*.** Metasoma with petiole hidden in dorsal view; gaster elongate, 2.6 times as long as broad, and as long or longer than head and mesosoma combined; scattered white setae at posterior margins of each tergite becoming more dense towards the apex of the gaster; each cercus with two long bristles. Ovipositor extends slightly beyond apex of gaster (Fig. 1A).

**MALE.** Length 1.2 – 1.6 mm. Antennae inserted in middle of face (Fig. 2C). Funicular segments each with 3 loosely ordered rows of fine bristles. Gaster ovate; shorter than head and thorax combined. Other characters similar to female.

##### Etymology.

Named after the ancient Egyptian god *Set*, whose mythological stories mirror the natural biology of *Euderus set*. *Set* was the god of evil and chaos (Pinch 2004) and was reported to have control over evil animals like hyenas and serpents, just as *Euderus set* manipulates the behavior of its host, which is a parasite of trees (see *Biology* section below). The god *Set* is also reported to have trapped his brother Osiris in a crypt to kill him, later retrieving the body and chopping it up into small piece, which also fits with *Euderus set*, the crypt-keeper wasp, which kills its host in a crypt, and devours the host from the inside out, leaving major sections of exoskeleton (i.e., body parts) chopped up and distributed in the crypt (Weinersmith et al., in revision).

##### Diagnosis.

Two changes are required for the new species, *Euderus set*, to be included in the North American key to species of the genus *Euderus* by Yoshimoto (1971). First, a change is required to the key to subgenera of *Euderus*, where characters referring to the male flagellum should be removed. Yoshimoto (1971) used whorls of long hairs on the male flagellum as a diagnostic trait to discriminate between subgenera *Neoeuderus* and *Euderus*, but at that time only a single male specimen of subgenus Neoeuderus was available for study, and this individual was from the species *Euderus viridilineatus* for which no females had been found. As the male *Euderus set*. do not have antennae with pronounced whorls of long hairs, this is not an appropriate diagnostic trait for the subgenus.

We propose the following revision to the Yoshimoto (1971) key to subgenera:

**Table:** 

4	Apical margin of fore wing with 5 hair lines; female antenna inserted at level of lower margin of eye; male antenna inserted about middle of face	***Neoeuderus***
–	Apical margin of fore wing with 3-4 hair lines (Fig. 2A); antenna of male and female inserted slightly above or at level of lower margin of eye (Fig. 2B, C)	***Euderus***

Secondly, Yoshimoto’s (1971) key to the subgenus Neoeuderus should be revised as follows:

**Table:** 

3	Neck region of stigmal vein short, stigmus large (1.0) with 6-10 scattered hairs on surface, postmarginal vein between 1.2 and 1.5 times length of stigma vein; radial cell with one or more scattered hairs	**4**
–	Neck of stigmal vein elongate, stigmus small (0.5), surface with 4 hairs; postmarginal vein 2 times length of stigma vein; radial cell bare	***multilineatus* (Girault)**
4	Surface of stigmus with 9-10 scattered hairs; submarginal vein with 7-8 dorsal bristles; 6 admarginal hairs; callus with 12 scattered hairs; apical region of radial cell with >1 scattered hairs	***crawfordi* Peck**
–	Surface of stigmus with 4–6 scattered hairs; submarginal vein with 6 dorsal bristles; 3–4 admarginal hairs; radial cell with 1 hair at approximately its median; (Fig. 2A); callus with 6-7 scattered hairs (Fig. 1A, C);	***set***

##### Molecular barcodes to complement morphological taxonomy.

The two female *Euderus set* mtDNA-COI sequences were 98% identical to each another and each was most similar to other previously identified *Euderus* in the BOLD database. Sequence 1 was 88.4% identical to *Euderus* sp. D0703 on BOLD and sequence 2 was 89.8% identical to another *Euderus* sp. on BOLD (Ratnasingham and Hebert 2007). See Supplemental File 1 for the two specific mtDNA sequences.

##### Distribution.

Type locality for *Euderus set* is Inlet Beach, Florida, U.S. (Lat/Long: 30.273663, -86.001911), where it emerged from a stem crypt gall on *Quercus geminata* induced by the crypt gall wasp, *Bassettia pallida*. We have also collected *Bassettia pallida* galls from live oaks across the Gulf coast of the southeastern United States, where additional *Euderus set* have been found, including additional sites in Georgia, Florida, Mississippi, Louisiana, and Texas (see Table 1). Generally, we expect *Euderus set* to be restricted to the range of live oaks (Quercus; subsection Virentes; Cavender-Bares et al. 2015) upon which *Bassettia pallida* induce galls (Melika and Abrahamson 2007).

##### Biology.

The genus *Euderus* Haliday is a small group of chalcidoid wasps belonging to the family Eulophidae (Burks 2003) where the majority are reported to be primary parasitoids of arthropods at all stages of development (Burks 1979, Burks 2003, Noyes 2016). *Euderus set* parasitizes the crypt gall wasp *Bassettia pallida* Ashmead, 1896, which forms galls on American live oaks in the genus *Quercus* and the subsection Virentes across the southeastern United States (Ashmead 1896, Melika and Abrahamson 2007, Egan et al. 2013). There are six to eight different cynipid gall wasps that are highly specialized and form galls on this same live oak complex (Egan et al. 2013). We have reared out many of the parasitoids from this large community, including clearly documenting the community from another gall former, *Belonocnema treatae* (Forbes et al. 2016), but this is the first time we have observed a member of the genus *Euderus* in this system after two decades of work (Lund et al. 1996, Egan and Ott 2007, Egan et al. 2012, Egan et al. 2013, Egan, unpubl. data).

Published records almost certainly underestimate the diversity of subgenus Neoeuderus in North America, and many other species in the subgenus may also be specialist parasitoids of oak galling cynipids. Yoshimoto (1971) noted that the biological records of the Nearctic *Euderus* indicate that most species are host specific, while also cautioning that host records for *Euderus* are patchy and the result of field observation, which only represent the most common species where there are substantial rearing records. While Yoshimoto noted just four members of subgenus Neoeuderus, the current work adds a fifth, and we have recently reared another from the honey comb leaf gall wasp, *Callirhytis favosa*, on pin oak in Iowa. While this undescribed *Euderus* parasitoid of *Callirhytis favosa* has not yet been extensively studied, it is most similar in appearance to *Euderus set*, *Euderus crawfordi*, and *Euderus multilineatus*. If this truly is a different species, then three of the six species in subgenus Neoeuderus are known parasitoids of the oak-associated Cynipidae.

Emergence of *Euderus set* in the lab from field-collected *Bassettia pallida* galls was concentrated from February to March coincident with new leaf growth of the host plants and adult maturation and emergence of the asexual generation *Bassettia pallida* (Melika and Abrahamoson 2007, Egan, unpubl. data). We also observed a smaller pulse in September and October, which could have been a natural occurrence, or induced by harvesting galled tissue and bringing it into a controlled environment. Regardless of harvest time (August or October), a similar emergence window was observed in February and March.

*Euderus set* is strongly associated with a behavioral phenotype in its host, the crypt gall wasp, *Bassettia pallida*, where infected gall wasps cut an emergence hole through the gall tissue as an adult, but then die and remain partially in the crypt to plug the emergence hole with its head (Weinersmith et al., in revision). When *Euderus set* emerges, it cuts an emergence hole directly through the head capsule plugging the hole (Weinersmith et al., in revision). The host’s behavioral phenotype may benefit *Euderus set* by making it easier for the adult stage to emerge from the crypt (as it now only has to emerge through the parasitoid’s head capsule, rather than through the tree stem itself; Weinersmith et al., in review). This putative behavioral manipulation of the host by its parasitoid *Euderus set* is the first time this has been described by the species-rich and economically important Chalcidoidea and is also the inspiration behind both the scientific name, *Euderus set*, and the common name, the crypt-keeper wasp.

In addition to *Euderus set*, we have also reared eleven additional natural enemy species from *Bassettia pallida* galls on live oaks (Quercus; subsection Virentes), including two inquilines (genera *Synergus* and *Ceroptres*) and nine parasitoids including three species from the genus *Sycophila*, two species from genus *Ormyrus*, one each from the genera *Eurytoma*, *Acaenacis*, and *Brasema*, as well as a parasitoid from the platygastrid subfamily Platygastrinae that we have not yet been able to key to genus. The natural enemy community requires further description.

## Supplementary Material

XML Treatment for Euderus

XML Treatment for Euderus set

## Supplementary materials

Supplementary material 1 Morphological, geographic, and ecological distinguishing features of *Euderus set* Data type: PDF File This dataset is made available under the Open Database License (http://opendatacommons.org/licenses/odbl/1.0/). The Open Database License (ODbL) is a license agreement intended to allow users to freely share, modify, and use this Dataset while maintaining this same freedom for others, provided that the original source and author(s) are credited.Scott P. Egan, Kelly L. Weinersmith, Sean Liu, Ryan D. Ridenbaugh, Y. Miles Zhang, Andrew A. Forbes
