# Supplementary material for: Description of a new species of Euderus Haliday from the southeastern United States (Hymenoptera, Chalcidoidea, Eulophidae): the crypt-keeper wasp
Source: Zookeys. 2017 Jan 12;(645):37–49. doi: 10.3897/zookeys.645.11117 (PMC5299223; doi:10.3897/zookeys.645.11117)
Supplement: Supplementary material 1 — Morphological, geographic, and ecological distinguishing features of Euderus set [file zookeys-645-037-s001.pdf]

Supplemental File 1

To compliment the main species description, we further describe morphological, geographic, and ecological distinguishing features of this new *Euderus* species, *Euderus set* sp. n*.,* which differentiate it from (1.) ecologically similar *Euderus* species attacking cynpid gall wasps, (2.) geographically overlapping *Euderus* species in the coastal southeastern United States, (3.) geographically proximate *Euderus* species from the Caribbean, and (4.) morphologically similar species within the *Euderus* genus. In addition, we provide DNA sequence data to complement the morphological taxonomy.

General Euderus morphology

The genus *Euderus* can be recognized from other genera in the Eulophidae by the presence of three or more hair lines radiating from the base of the stigma1 vein, by the postmarginal vein in the forewing, and by complete notaulices (Yoshimoto 1971). Yoshimoto (1971) described five subgenera within the genus *Euderus* (*Euderoides* subgen., *Neoeuderus* subgen., *Leipocrossus* subgen., *Secodelloidea* Girault subgen., and *Euderus* Haliday s. str.) and provides a key to subgenera and species. Among these five subgenera, *Euderus*, *Neoeuderus*, and *Leipocrossus* share a number of common and distinguishing characters from the other two subgenera, *Euderoides* and *Secodelloidea*, including features of the antennae, mesosoma, and fore and hind wings (see Table 2 in Yoshimoto 1971).

Distinguishing features from ecologically similar Euderus species that attack cynipids

Only two *Euderus* species have been reported to attack members of the Cynipidae: *Euderus albitarsis* (Zetterstedt 1838) and *E. crawfordi* (Peck 1951). Interestingly, both cynipid-*Euderus* associations from this cosmopolitan genus are reported from the U.S.

The cosmopolitan species *E. albitarsis* is found in Europe, Asia, including India, Australia, and North America. In North America, *E. albitarsis* has been reported from the U.S. states of West Virginia and Virginia, as well as Canada, where it was reported to attack an unidentified cynipid (Bouček and Askew 1968, Yoshimoto 1971**)**. *E. albitarsis* is placed within the subgenus *Euderus*, whereas the new species, *Euderus set sp. n.,* described herein, is placed in subgenus *Neoeuderus*. Members of the subgenus *Neoeuderus* can be distinguished from other subgenera by the presence of five hair lines on the apical margin of the forewing, and by a combination of other sex-specific differences, including female antenna inserted at the level of the lower margin of eye, while males have antenna inserted at about the middle of the face (Yoshimoto 1971).

The second *Euderus* species associated with cynipids is *E. crawfordi*, which was reported to attack the cynpids *Dryocomus coxii* and *Plagiotrochus suberi* on the host plant *Quercus suber* in the California and Arizona (Burks 1979, Zuparko 1996). *E. crawfordi* and *E. set sp. n.* are from the same subgenus *Neoeuderus*, and while they are morphologically similar, several distinguishing morphological, ecological, and geographic characters can be observed. These differences are described in the sections "*Distinguishing features from morphologically similar species*" and "*Diagnosis*", which includes the revised couplets to the Yoshimoto (1971) key.

Distinguishing features from geographically overlapping species from the coastal southeastern U.S.

Based on this revision, there are 12 *Euderus* reported from the coastal southeastern U.S. (Florida, Alabama, Mississippi, Louisiana, and Texas), of which 10 species are reported from the state of Florida (Yoshimoto 1971, Noyes 2016), where *Euderus set sp. n.*, was originally discovered and is the type locality. Of the 12 species reported from this region, only one species, *E. multilineatus*, falls within the subgenus *Neoeuderus* containing *Euderus set sp. n.* Interestingly, Yoshimoto (1971) notes that *E. multilineatus* is quite similar to *E. crawfordi*, which is one of the two species of *Euderus* reported to attack cynpid gall wasps (Burks 1979, Zuparko 1996). *E. multilineatus* and *E. crawfordi* can be distinguished from other members of the subgenus *Neoeuderus* by (1.) an antennal scape that is dark metallic green rather than yellow to yellowish orange, (2.) a gaster that is elongate-ovate rather than a short and broad gaster that is conic-ovate, and (3.) a propodeal callus that has 12 or 6 long white hairs, rather than 4 long hairs (Yoshimoto 1971). *E. multilineatus* is reported from Texas (Girault 1917, Peck 1963), however has no reported host associations (Yoshimoto 1971, Noyes 2016). *Euderus multilineatus, E. crawfordi* and *E. set sp. n.* are from the same subgenus *Neoeuderus*, where they are morphologically similar, but several distinguishing characters, including morphological, ecological, and geographic can be observed. These differences are clearly described in the section below "*Distinguishing features from morphologically similar species*" and in the revised couplet to Yoshimoto (1971).

Of the other eleven *Euderus* species within the coastal southeastern United States, seven are from the subgenus *Euderus* and one each are from the subgenera *Leipocrossus*, *Euderoides*, and *Secodelloidea*. There was also one other species from the genus *Euderus* found in Florida, *E. elongatus*, that remained unplaced by Yoshimoto (1971) and Burks (1970, pers. comm. as reported in Yoshimoto 1971) due to damaged type specimens. This unplaced species, *E. elongatus*, is reported from Connecticut, Maine, New Hampshire, and Florida, where it was reported to parasitize *Attelabus rhois* (Coleoptera: Attelabidae) on the host plant *Ambrosia artemisiifolia* (Asteraceae) (Ashmead 1887, Peck 1963, Burks 1979).

Distinguishing features from geographically proximate species from the Caribbean

We also considered *Euderus* from the Caribbean given that members of the Chalcidoidea from this region can also be found in Florida. First, using the Chalcidoidea Database (Noyes et al. 2016) and a literature search, we found no record of *Euderus* attacking Cynipidae in this region. Moreover, *Euderus set* appears to be a specialist on the crypt galler *Bassettia pallida* in the southeastern United States, which itself is a specialist on the live oaks *Q. virginiana* and *Q. geminata* that are only distributed along the southeastern U.S. along the Gulf coast. Based on biology, there appears to be no direct match to *E. set*.

We did find four *Euderus* species recorded in the Caribbean, *E. lividus*, *E. pallidiscapus*, *E. striata*, and *E. varicolor*.

E. lividus was reported in North America (Ashmead 1886) and the West Indies (Puerto Rico; Herting 1975) and was considered by Yoshimoto (1971), where he grouped *E. lividus* within a different subgenus than *E. set* (subgenus *Euderus*) based on morphology.

E. pallidiscapus was excluded based on morphology in the original species description (Gahan 1934) and natural history, where it is reported to attack Coleoptera and Diptera in Cuba (De Santis 1979).

E. varicolor was excluded based on morphology in the species description (Riley et al. 1894), subsequent revision (LaSalle and Shauff 1992), and reported geographic distribution in Puerto Rico, St. Vincent and Grenadines, and Grenada (De Santis 1979).

E. striata was excluded based on morphology in the species description (Howard 1897), subsequent revision (LaSalle and Shauff 1992), and reported geographic distribution in Grenada (Howard 1897, DeSantis 1979).

Distinguishing features from morphologically similar species

Based on the most recent taxonomic key for *Euderus* (Yoshimoto 1971), a species fitting the exact morphology of *Euderus set sp. n.* does not appear, nor does a species that matches its host association in this same geographic region (Noyes 2016). Using Yoshimoto (1971), *E. set sp. n.* keys out to the subgenus *Neoeuderus*, and the closest species is *E. crawfordi*, which is interesting, since *E. crawfordi* is one of two species of *Euderus* reported to parasitize cynipid gall wasps. However, there are clear morphological, geographic and ecological differences. Most notably, the two species differ in the number of hairs on their callus (12 in *E. crawfordi*, 6-7 in *E. set sp. n.*), and several fore wing characters, including the number of admarginal hairs, the number of bristles on the submarginal vein, and the number of hairs on the stigmus. Further details are provided in the revised couplet to the Yoshimoto (1971) key below. Moreover, as we document above, *E. crawfordi* exhibits (1.) no geographic overlap, where it is reported from roughly 1300km outside the geographic range *E. set* sp. n., (2.) no ecological overlap, where *E. crawfordi* is associated with an oak species from a different section within the large genus *Quercus* (*E. crawfordi* is associated with the section *Cerris*; whereas *E. set* is associated with live oaks in the section *Quercus*), and (3.) no host prey overlap, where *E. crawfordi* has been reported to attacking cynipids from different genera than *E. set sp. n.* (Burks 1979, Zuparko 1996). The new species also differs from the geographically overlapping species, *E. multilineatus*. These two species differ in the number of hairs in their radial cells and on their stigma, and in the relative lengths of their postmarginal and stigmal veins.

Survey of new species described since the Yoshimoto (1971) revision

To confirm that *Euderus set sp. n.* has not been described since Yoshimoto (1971), we surveyed the literature for new *Euderus* species described since the publication of Yoshimoto (1971). To do this, we searched the scientific literature using Google Scholar and Web of Science with the keywords '*Euderus*' and 'new species' in papers published between 1971 and the present (December 2016). We also searched the Universal Chalcidoidea Database maintained by the Natural History Museum - London for species described since 1971 (Noyes 2016).

We found seven species of *Euderus* described since Yoshimoto (1971). All were described from type localities outside of North America and none were found to be associated with cynipid gall wasps or their oak host plants (*Quercus*; subsection *Virentes*). Original species descriptions were examined and none fit with the new species described here. Each species is listed alphabetically, followed by known distributions, primary hosts (if known), and plant associates (if known).

Euderus alcidodes Singh, 2005
Distribution: Assam, India
Primary hosts: *Alcidodes ludificator* (Coleoptera; Curculionidae)
Plant associates: *Gmelina arborea* (Verbenaceae)

Euderus alvarengai De Santis & Diaz, 1975
Distribution: Fernando do Noronha, Brazil
Primary hosts: there are no associates listed for this taxon
Plant associates: there are no associates listed for this taxon

Euderus beijingensis (Luo & Liao, 1985)
Distribution: Beijing, China
Primary hosts: there are no associates listed for this taxon
Plant associates: there are no associates listed for this taxon

Euderus fasciatus Askew, 2001
Distribution: Spain
Primary host: there are no associates listed for this taxon
Plant associates: *Gypsophila struthium* (Caryophyllaceae), *Rosmarinus officinalis* (Lamiaceae)

Euderus lindemani Fursov, 1997
Distribution: Kazakhstan (Astana), Russia (Astrakhan' Oblast)
Primary hosts: *Scolytus kirschi* (Coleoptera; Scolytidae)
Plant associates: there are no associates listed for this taxon

Euderus regiae Yang, 1996
Distribution: Shaanxi, China
Primary host: *Zyloborus sp.* (Coleoptera; Scolytidae)
Plant associates: *Juglans regia* (Juglandaceae)

Euderus viridulus Bouček, 1988
Distribution: Australia (Queensland), South Africa
Primary hosts: *Gonipterus scutellatus* (Coleoptera; Curculionidae)
Plant associates: there are no associates listed for this taxon

Molecular barcodes to complement morphological taxonomy

The two female *Euderus set sp. n.* COI sequences were 98% identical to each another and each was most similar to other previously identified *Euderus* in the BOLD database. Sequence 1 was

88.4% identical to *Euderus sp.* D0703 on BOLD and sequence 2 was 89.8% identical to another *Euderus sp.* on BOLD (Ratnasingham and Hebert 2007).

Euderus set COI sequence 1:
AATTACTTAGTTCCTATAATTTTAGGGAGCCCAGATATAGCATTTCCTCGAATAAAT
AATATAAGGTTTTGATTACTTCCCCCTAGAATTATTTTATTAATTAGAAGAATATTTA
TTGGAACTGGGACTGGAACTGGATGAACTGTTTATCCTCCTTTATCAAGAAATTTAT
CTCATAGTGGGCCATCTGTTGATTTATCAATTTTTTCTTTACATATTGCGGGGGTTTC
TTCTATTATAGGTTCAATTAATTTTATTACCACTATTTTAAATATAAAAATTTTTAAA
ATTGAATTAATTCCTTTATTTGCATGGGCTATATTATTAACTGCTATTTTACTACTTTT
ATCTCTTCCAGTATTAGCAGGAGCTATTACTATGCTATTATTTGATCGAAATTTAAAT
ACTTCATTTTTTGACCCCTCTGGAGGGGGGGACCCTATTTTATACCAACATTTATTTT
GATTTTTTGGACATCCTGAAGTTTATATTTTAATTCTACCTGGATTTGGCTTAGTTTCT
CATATAATTTGTAATGAAAGAATAAAAAAAGAGGTATTTGGATCTTTGGGAATAATT
TATGCAATAATTTCTATTGGTTTATTAGGATTTATTGTTTGAGCTCATCATATATTTA
CAGTAGGAATGGACGTAGATACTCGGGCATATTTTACTTCAGCTACNATAATTATTG
CCGTTCCAA

Euderus set COI sequence 2:
ATTTTTTTTTTTGTTATACCTGTAATAATAGGGGGATTTGGAAATTATTTAGTTCCTA
TAATTTTAGGAAGTCCAGATATAGCATTTCCTCGAATAAATAATATAAGGTTTTGAT
TACTTCCCCCTAGAATTATTTTATTAATTAGAAGAATATTTATTGGAACCGGGACTG
AACTGGATGAACTGTTTATCCTCCTTTATCAAGAAATTTATCTCATAGTGGGCCATCT
GTTGATTTATCAATTTTTTCTTTACATATTGCGGGAATTTCTTCTATTATAGGTTCAAT
TAATTTTATTACCACTATTTTAAATATAAAAATTTTTAAAATTGAATTAATTCCTTTA
TTTGCATGAGCTATATTATTAACTGCTATTTTATTACTTTTATCTCTTCCAGTATTAGC
AGGGGCTATTACTATGCTATTATTTGATCGAAATTTAAATACTTCATTTTTTGATCCC
TCTGGAGGGGGGGACCCTATTTTATATCAACATTTATTTTGATTTTTTGGACATCCTG
AAGTTTATATTTTAATTCTACCTGGATTTGGCTTAGTTTCTCATATAATTTGTAATGA
AAGAATAAAAAAAGAGGTATTTGGATCTTTAGGAATAATTTATGCAATAATTTCTAT
TGGTTTATTAGGATTTATTGTTTGAGCTCATCATATATTTACAGTAGGAATGGACGTA
GATACTCGGGCATATTTTACTTCAGCTACAATAATTATTGCCGTTCCAA

Supplemental File References

Ashmead WH (1886) Studies on the North-American Chalcididae with descriptions of new species from Florida. Transactions of the American Entomological Society 13:135.

Ashmead WH (1887) Studies on the North American Chalcididae, with descriptions of new species, chiefly from Florida. Transactions of the American Entomological Society 14: 183-203.

Bouček Z, Askew RR (1968) Hym. Chalcidoidea. Palearctic Eulophidae (excl. Tetrastichinae). Index of Entomophagous Insects 3: 72.

Burks BD (1979) Torymidae (Agaoninae) and all other families of Chalcidoidea (excluding Encyrtidae). In: Krombein, K.V.; Hurd, P.D. Jr.; Smith, D.R.; Burks, B.D., Editors, Catalog of Hymenoptera in America North of Mexico, Smithsonian Institute Press, Washington, D.C.

De Santis L (1979) Catálogo de los himénopteros calcidoideos de América al sur de los Estados Unidos. *Publicación Especial Comisión de Investigaciones Cientificas Provincia de Buenos Aires* pp.266.

Gahan AB (1934) Descriptions of some new species of Chalcidoidea from Cuba and Puerto Rico. *Memorias de la Sociedad Cubana de Historia Natural, Habana* 8:133.

Herting B (1975) Lepidoptera, Part 1 (Microlepidoptera). A catalogue of parasites and predators of terrestrial arthropods. Section A. Host or Prey/Enemy. Commonwealth Agricultural Bureaux, Commonwealth Institute of Biological Control 6:22–24.

Girault AA (1917) Three new chalcid flies from North America. Bulletin of the Brooklyn Entomological Society 12: 85-86.

Howard LO (1897) On the Chalcididae of the Island of Grenada. Journal of the Linnean Society (Zoology) 26:165.

LaSalle J, Schauff ME (1992), Preliminary studies on neotropical Eulophidae (Hymenoptera: Chalcidoidea): Ashmead, Cameron, Howard and Walker species. Contributions of the American Entomological Institute 27(1) pp. 47pp.

Riley CV, Ashmead WH, Howard LO (1894) Report upon the parasitic Hymenoptera of the island of St. Vincent. Journal of the Linnean Society (Zoology) 25:168.

Noyes JS (2016) Universal Chalcidoidea Database. World Wide Web electronic publication. http://www.nhm.ac.uk/chalcidoids.

Peck O (1951) Superfamily Chalcidoidea. In: Muesebeck, C.F.W., Krombein, K.V. & Townes, H.K. (Editors). Hymenoptera of America north of Mexico - synoptic catalog. Agriculture Monographs. U.S. Department of Agriculture. 2: 462.

Peck O (1963) Catalogue of Nearctic Chalcidoidea (Insecta: Hymenoptera). Can. Ent. Suppl. 30: 197-201.

Ratnasingham S, Hebert PDN (2007) BOLD: The barcode of life data system. Molecular Ecology Notes 7: 355-364.

Yoshimoto CM (1971) Revision of the genus *Euderus* of America north of Mexico. Canadian Entomologist 103: 541-578.

Zetterstedt JW (1838) Hymenoptera. Insecta Lapponica 1: 423-428.

Zuparko RL (1996) Hymenoptera reared from *Plagiotrochus suberi* (Hymenoptera: Cynipidae) galls in California. Pan-Pacific Entomologist 72: 27-30.

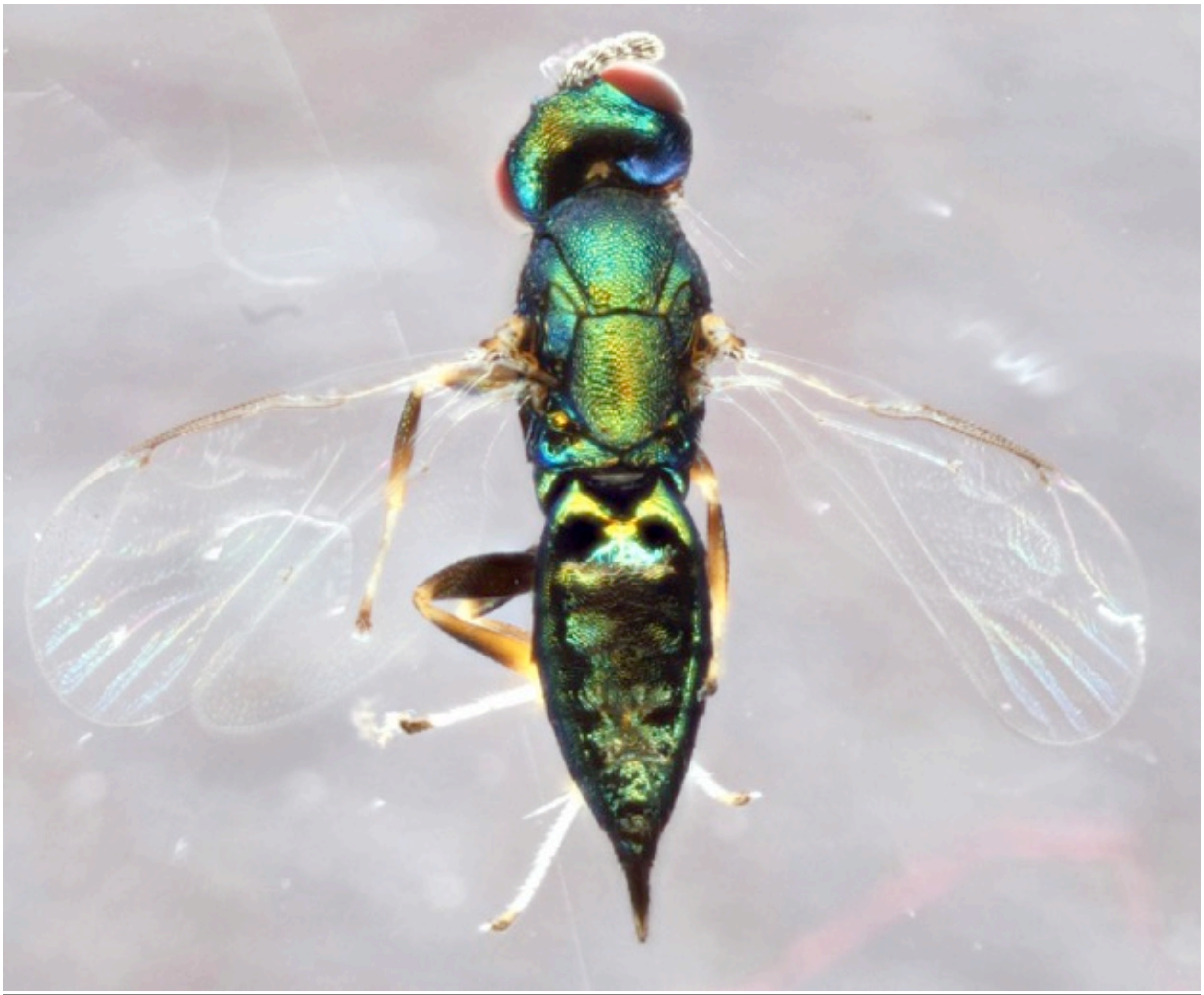

Supplemental Figure 1. Dorsal habitus of female *Euderus set.*
